# ROCS: A reproducibility index and confidence score for interaction proteomics

**DOI:** 10.1186/1471-2458-13-1009

**Published:** 2013-11-12

**Authors:** Allen Kabagenyi, Patricia Ndugga, Stephen OJIAMBO Wandera, Betty Kwagala

## Abstract

Background Affinity-Purification Mass-Spectrometry (AP-MS) provides a powerful means of identifying protein complexes and interactions. Several important challenges exist in interpreting the results of AP-MS experiments. First, the reproducibility of AP-MS experimental replicates can be low, due both to technical variability and the dynamic nature of protein interactions in the cell. Second, the identification of true protein-protein interactions in AP-MS experiments is subject to inaccuracy due to high false negative and false positive rates. Several experimental approaches can be used to mitigate these drawbacks, including the use of replicated and control experiments and relative quantification to sensitively distinguish true interacting proteins from false ones. Results To address the issues of reproducibility and accuracy of protein-protein interactions, we introduce a two-step method, called ROCS, which makes use of Indicator Proteins to select reproducible AP-MS experiments, and of Confidence Scores to select specific protein-protein interactions. The Indicator Proteins account for measures of protein identification as well as protein reproducibility, effectively allowing removal of outlier experiments that contribute noise and affect downstream inferences. The filtered set of experiments is then used in the Protein-Protein Interaction (PPI) scoring step. Prey protein scoring is done by computing a Confidence Score, which accounts for the probability of occurrence of prey proteins in the bait experiments relative to the control experiment, where the significance cutoff parameter is estimated by simultaneously controlling false positives and false negatives against metrics of false discovery rate and biological coherence respectively. In summary, the ROCS method relies on automatic objective criterions for parameter estimation and error-controlled procedures. We illustrate the performance of our method by applying it to five previously published AP-MS experiments, each containing well characterized protein interactions, allowing for systematic benchmarking of ROCS. We show that our method may be used on its own to make accurate identification of specific, biologically relevant protein-protein interactions or in combination with other AP-MS scoring methods to significantly improve inferences. Conclusions Our method addresses important issues encountered in AP-MS datasets, making ROCS a very promising tool for this purpose, either on its own or especially in conjunction with other methods. We anticipate that our methodology may be used more generally in proteomics studies and databases, where experimental reproducibility issues arise. The method is implemented in the R language, and is available as an R package called "ROCS", freely available from the CRAN repository http://cran.r-project.org/.

## Methods

We employed our newly adopted SVM method in the continuous measurement of the cortical width of the mandible on dental panoramic radiographs to identify osteoporosis. We compared the diagnostic performances between this newly developed method and our trimmed mean method in identifying women with low BMD at the lumbar spine and the femoral neck in 100 postmenopausal women ([greater than or equal to]50 years), of whom 60 to the training and 40 to its testing with no previous record of osteoporosis. Cortical width below the mental foramen of the mandible on dental panoramic radiographs was measured continuously with CAD system by enhancing the original image of the X-ray, determining cortical boundaries, evaluating all the distances among upper and lower boundaries and finally discriminating by radial basis function - support vector machine method.

## Results and Discussion

The sensitivity and specificity of the cortical measurements of the lumbar spine were 90.9% (95% confidence interval shown in parentheses) (85.3-96.5) and 83.8% (76.6-91.0), respectively and 90.0% (84.1-95.9) and 69.1% (60.1-78.6), respectively with femoral neck BMD. In addition, the sensitivity and specificity for the combination of data of both the lumbar spine and femoral neck for identifying women with low BMD were 90.6% (92.0-100) and 80.9% (71.0-86.9), respectively. We assessed that the diagnosis and classification of women using support vector machine employing the average and variance of the continuous measurements provide excellent discrimination ability in comparison with the estimation of cortical width using the trimmed mean method. Conclusion: Results showed that our newly developed CAD system with SVM method improves the overall performance of the identification of high risk group of osteoporotic patients. Overall, most promising results in terms of best overall response rate (BORR) were obtained with 10 mg/kg of ipilimumab, every 3 weeks for a total of 4 doses (induction phase) followed by maintenance period in which ipilimumab was administrated every 12 weeks (maintenance phase). This was the reason for the choice of such a schedule for the front line phase 3 study. The most common treatment-related adverse events (AEs) associated with the use of ipilimumab were immune-related and specific algorithms have been subsequently developed, showing that early recognition and correct therapeutic approach with steroid therapy make most of these AEs manageable and reversible 

## Pre-publication history

The pre-publication history for this paper can be accessed here:

http://www.biomedcentral.com/1471-2458/13/1009/prepub

